# Bicuspidalization of the Native Tricuspid Aortic Valve: A Porcine in Vivo Model of Bicuspid Aortopathy

**DOI:** 10.3400/avd.oa.21-00116

**Published:** 2022-03-25

**Authors:** Naoyuki Kimura, Ryo Itagaki, Masanori Nakamura, Alimuddin Tofrizal, Megumi Yatabe, Takamichi Yoshizaki, Ryo Kokubo, Shuji Hishikawa, Satoshi Kunita, Hideo Adachi, Yoshio Misawa, Takashi Yashiro, Koji Kawahito

**Affiliations:** 1 Department of Cardiovascular Surgery, Saitama Medical Center, Jichi Medical University, Saitama, Saitama, Japan; 2 Department of Surgery, Division of Cardiovascular Surgery, Jichi Medical University, Shimotsuke, Tochigi, Japan; 3 Department of Electrical and Mechanical Engineering, Nagoya Institute of Technology, Nagoya, Aichi, Japan; 4 Department of Anatomy, Division of Histology and Cell Biology, Jichi Medical University, Shimotsuke, Tochigi, Japan; 5 Department of Medical Engineering, Saitama Medical Center, Jichi Medical University, Saitama, Saitama, Japan; 6 Medical Education and Training Core, Center for Development of Advanced Medical Technology, Jichi Medical University, Shimotsuke, Tochigi, Japan; 7 Animal Resource Laboratory, Center for Development of Advanced Medical Technology, Jichi Medical University, Shimo tsuke, Tochigi, Japan

**Keywords:** bicuspid aortic valve, bicuspidalization, pig, wall shear stress

## Abstract

**Objective**: To examine early histologic changes in the aorta exposed to bicuspid flow.

**Material and Methods**: A porcine bicuspid aortopathy model was developed by suturing aortic cusps. Of nine pigs, eight underwent sham surgery (n=3) or bicuspidalization (n=5); one was used as an intact control. Wall shear stress (WSS) was assessed by computational fluid dynamics (CFD). Animals were exposed to normal or bicuspid flow for 48 h and were then euthanized for histologic examinations.

**Results**: No animal died intraoperatively. One animal subjected to bicuspidalization died of respiratory failure during postoperative imaging studies. Echocardiography showed the aortic valve area decreased from 2.52±1.15 to 1.21±0.48 cm^2^ after bicuspidalization, CFD revealed increased maximum WSS (10.0±5.2 vs. 54.0±25.7 Pa; P=0.036) and percentage area of increased WSS (>5 Pa) in the ascending aorta (30.3%±24.1% vs. 81.3%±13.4%; P=0.015) after bicuspidalization. Hematoxylin–eosin staining and transmission electron microscopy showed subintimal edema and detached or degenerated endothelial cells following both sham surgery and bicuspidalization, regardless of WSS distribution.

**Conclusion**: A bicuspid aortic valve appears to increase aortic WSS. The endothelial damage observed might have been related to non-pulsatile flow (cardiopulmonary bypass). Chronic experiments are needed to clarify the relationship between hemodynamic stress and development of bicuspid aortopathy.

## Introduction

Bicuspid aortic valve (BAV) is one of the most common congenital heart valve anomalies, with recent large-scale studies showing the prevalence of BAV in the general population to range between 0.43% and 0.77%.^1,2)^ Dilatation of the proximal thoracic aorta, or bicuspid aortopathy, occurs in up to 50% of adult patients with BAV.^3,4)^ The pathogenesis of BAV-associated aortopathy is complex, and the underlying mechanisms remain to be elucidated. Various studies have been conducted to investigate its pathogenesis, with examination of aortic specimens from patients with bicuspid aortopathy having been a standard research approach.^5–7)^ However, aortic specimens can be harvested only from patients whose aorta is pathologically dilated. Thus, clinical investigation into the early histologic and hemodynamic changes characteristic of bicuspid aortopathy is difficult. Transgenic mice have been used to create in vivo models of bicuspid aortopathy,^8,9)^ with congenital BAV being quite rare in large animals,^10,11)^ and there have been no reports of use of large animals as in vivo experiment model. To investigate early changes in the aortic wall that occur in response to eccentric flow jets created by BAV, we established a porcine in vivo bicuspidalization model. Aortic hemodynamics associated with bicuspidalization was assessed by computational fluid dynamics (CFD) simulation, and early bicuspidalization-associated histologic changes in different areas of the thoracic aorta were also assessed.

## Materials and Methods

### Overview

This study was conducted at an accredited animal research laboratory (Center for Development of Advanced Medical Technology, Jichi Medical University, Tochigi, Japan). Approval was obtained from the Animal Experiment Committee of Jichi Medical University prior to its execution (authorization no. 15230). All study procedures, other than CFD simulation, were performed at Jichi Medical University. Eight female miniature (crossbred KCG or Mexican hairless) pigs (age: 8–14 months; average weight: 45.8±2.6 kg) were obtained from the National Livestock Breeding Center, Ibaraki Station (Ibaraki, Japan), and one female domestic pig (age: 3 months; weight: 41.0 kg) was obtained from Sanesu Breeding Co., Ltd. (Chiba, Japan). The study schema is shown in Supplementary **Fig. 1**. Eight of the nine pigs were divided between a sham surgery group (n=3, negative control) and a bicuspidalization group (n=5), and one was set aside as an intact control. All pigs were acclimatized for a minimum of 6 days, and those in the sham surgery and bicuspidalization groups were fasted for 24 h with free access to water prior to the surgical procedure. Computed tomography (CT) and magnetic resonance imaging (MRI) were performed 48 h after the surgical procedure. After 48 h of exposure of the tricuspid valve (in pigs subjected to sham surgery) and of the bicuspidalized valve to blood flow, the animals were euthanized by intravenous infusion of potassium chloride under sevoflurane anesthesia. Aortic tissue specimens were obtained immediately thereafter for histologic examination. CT and MRI were also performed in the intact control animal, and that animal was fasted for 24 h and then euthanized in the same manner as the other animals. Aortic tissue specimens were also obtained from that animal.

### Surgical procedure

The eight pigs subjected to surgery were premedicated by intramuscular injection of midazolam (0.3 mg/kg), medetomidine (0.06 mg/kg), and atropine (0.02 mg/kg). On confirmation of sedation, the pigs were endotracheally intubated. General anesthesia was induced and maintained by inhalation of 2%–5% sevoflurane. A 20G catheter was placed in an ear vein for venous access, and 10 mg succinylcholine was administered for muscle relaxation. The pigs were then placed in the supine position. The right side of the neck was incised, and a central venous catheter was inserted into the right external jugular vein. To establish cardiopulmonary bypass (CPB), a 16F arterial cannula was placed in the right carotid artery, and a 26F venous cannula was inserted into the superior vena cava via the right internal jugular vein. A 5F sheath was inserted into the right femoral artery, and this arterial line was then connected to the FloTrac sensor on the EV 1000 platform (Edwards Lifesciences, Irvine, CA, USA) for monitoring the following hemodynamic variables: blood pressure, pulse rate, cardiac output, cardiac index, stroke volume, systemic vascular resistance, and central venous pressure. Rectal temperature and electrocardiogram signals were measured continuously throughout the surgical procedure.

The pigs were next placed in the right lateral decubitus position. A skin incision was made on the left chest, left thoracotomy through the third intercostal space was performed, and the pericardium was incised. After achievement of systemic heparinization (3 mg/kg) at a target activated clotting time of >500 seconds, a 26 F venous cannula was inserted into the inferior vena cava via the right atrium for bicaval venous cannulation. A venting tube was inserted into the left ventricle via the left atrial appendage, and CPB was begun. The animals were not cooled during CPB. The ascending aorta was clamped, and cardiac arrest was achieved by antegrade infusion of 100–150 mL cold blood cardioplegia solution consisting of 250 mL Ringer’s lactate solution plus 40 mL potassium chloride. One of the following two types of bicuspidalization was performed: joining of the right coronary cusp and the noncoronary cusp (RN bicuspidalization) or joining of the left coronary cusp and the noncoronary cusp (LN bicuspidalization), both with running 6-0 polypropylene suture. Fusion of the right and left coronary cusps was not simulated. Sham surgery consisted of aortotomy (Supplementary **Fig. 1**), and the incision was closed with running 4-0 polypropylene suture. The ascending aorta was de-aired and then declamped. If ventricular fibrillation occurred after declamping, electrical cardioversion was performed to restore the heart’s regular rhythm. Low-dose dopamine was used to reduce the risk of acute kidney injury. Once hemostasis was confirmed, drainage tubes were placed in the pericardium and left thoracic cavity. The muscular layer was closed with running 1-0 vicryl, and the skin and subdermal tissue were closed with 3-0 nylon mattress sutures. Left ventricular function and degree of aortic stenosis after bicuspidalization were evaluated intraoperatively by transthoracic echocardiography. A cell-saver device was used to collect blood lost before and after CPB. Acetated Ringer’s solution was infused to replace fluid lost during surgery. To prevent surgical site infection, 1 g cefazolin was administered intraoperatively. For postoperative pain relief, buprenorphine hydrochloride was injected intraoperatively, and buprenorphine transdermal patches were applied postoperatively.

### CFD simulation of aortic blood flow

CFD simulation of aortic blood flow was performed in the manner previously described among patients with a BAV.^12)^ Details are shown in the Supplementary Material.

### Histologic examination

Histologic examination was conducted in blinded fashion by two histologists (A.T. and M.Y.) on thoracic aorta tissues obtained at the following three sites: (1) the greater curvature of the proximal ascending aorta, (2) the lesser curvature of the proximal ascending aorta, and (3) the proximal descending aorta. Both light microscopy examination and transmission electron microscopy (TEM) examinations were performed. For light microscopy examination, harvested tissues were fixed in 4% paraformaldehyde in 0.1 M phosphate buffer (pH 7.4) for 24 h. Tissues were then dehydrated in a graded series of alcohols, cleared with xylene, and embedded in paraffin. The paraffin blocks were sectioned at 4-µm thickness with a rotary microtome, and the sections were stained with hematoxylin and eosin (H&E).

For TEM, the harvested tissues were cut into small pieces with a razor blade and fixed with 2.5% glutaraldehyde in 0.1 M phosphate buffer (pH 7.4) containing 4% sucrose at 4°C for 2 h. The tissues were washed with 0.1 M phosphate buffer (pH 7.2) containing 4% sucrose, fixed with 1% OsO_4_ in 0.1 M phosphate buffer (pH 7.4) at 4°C for 90 min, dehydrated in an ethanol series, and embedded in Quentol-812 epoxy resin. Ultrathin sections were prepared, stained with 2% uranyl acetate and Reynold’s solution, then examined under a Hitachi H-7600 transmission electron microscope.

### Statistical analysis

Continuous variables are shown as mean±standard deviation, and categorical variables are shown as the number (percentage) of animals. Differences in variables between animals subjected to bicuspidalization and sham surgery were compared using the chi-square or Student t-tests. All statistical analyses were performed with IBM SPSS Statistics 26.0 for Windows (IBM Corp., Armonk, NY, USA), and P<0.05 was considered significant.

## Results

Basic characteristics, operative variables, and surgical outcomes are shown for all eight animals subjected to either sham surgery or bicuspidalization and per group in Supplementary **Table 1**. The body weight and preoperative left ventricular ejection fraction did not differ between the bicuspidalization and the sham surgery groups. Operative variables also did not differ between the groups. All animals were weaned from CPB and none died intraoperatively. No animal suffered postoperative neurologic complications. One animal subjected to LN bicuspidalization died of respiratory failure 48 h after the surgery during induction of anesthesia for the CT and MRI examinations. Unfortunately, echocardiography data were not saved for this animal. For the four remaining animals subjected to bicuspidalization (RN, n=2; LN, n=2), echocardiography showed the pre- and post-bicuspidalization aortic valve area to be 2.52±1.15 and 1.21±0.48 cm^2^, respectively. A representative echocardiogram obtained after LN bicuspidalization is shown in Supplementary **Video 1**. The LN bicuspidalization resulted in a decreased aortic valve area.

**Figure 1:**
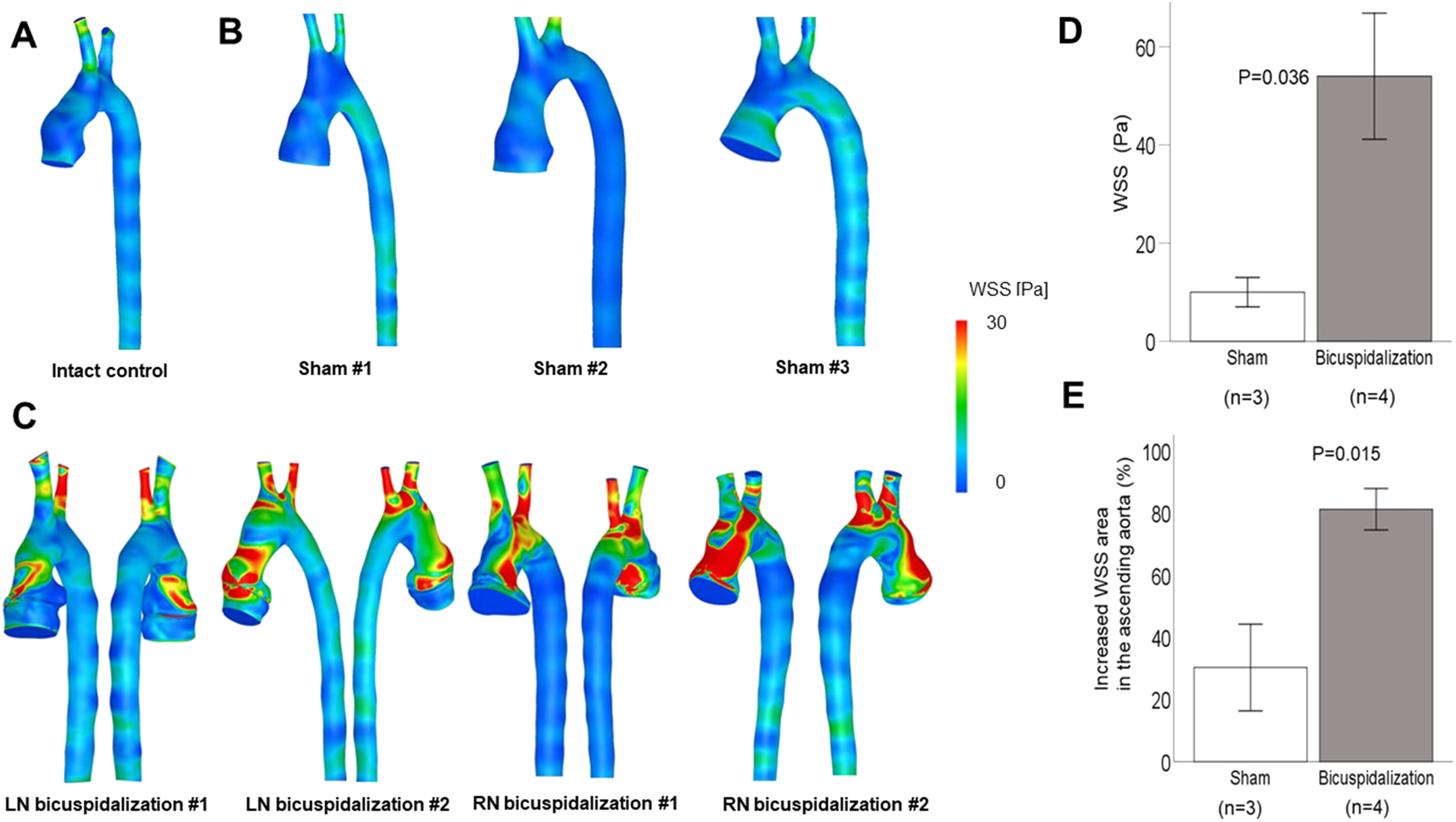
Fig. 1 CFD contour plots of WSS at peak systole, and bar graphs comparing WSS data between the sham surgery and bicuspidalization groups. (**A**) Simulation for the intact control animal (anterior view). (**B**) Simulation for the three animals subjected to sham surgery (anterior view). (**C**) Simulation for the four animals subjected to bicuspidalization (anterior and posterior views). (**D**) Bar graph of maximum WSS in the sham surgery and bicuspidalization groups. (**E**) Bar graph of the percentage of increased WSS (WSS >5 Pa) in the ascending aorta in the sham surgery and bicuspidalization groups.

CFD: computational fluid dynamics; WSS: wall shear stress; LN: left coronary cusp-noncoronary cusp; RN: right coronary cusp-noncoronary cusp; Pa: pascal

We used three-dimensional-CT imaging data and phase-contrast MRI measurements for CFD simulation of blood flow in eight of the nine animals (intact control, n=1; sham surgery, n=3; and bicuspidalization, n=4); the animal that died during induction of anesthesia was excluded. Contour plots of wall shear stress (WSS) at systole are shown for all eight animals in **Fig. 1**. CFD simulation revealed slightly increased WSS in the supra-arch vessels in the intact control animal (**Fig. 1A**). WSS in the animals subjected to sham surgery did not differ from that of the intact control animal (**Fig. 1B**). In contrast, WSS was shown on anteroposterior and posteroanterior views to be increased in the ascending aorta and supra-arch vessels of each of the four animals subjected to bicuspidalization (**Fig. 1C**). In the two animals subjected to LN bicuspidalization, WSS was greatest at the anterior wall and greater curvature of the proximal ascending aorta. In the two animals subjected to RN bicuspidalization, WSS was greatest at the anterior wall and lesser curvature of the proximal ascending aorta. Maximum WSS and percentage surface area of increased WSS (>5 Pa) in the ascending aorta are shown in **Figs. 1D** and **1E**, respectively, for the sham surgery and bicuspidalization groups. Both maximum WSS and percentage surface area of increased WSS were significantly greater in the bicuspidalization group than in the sham surgery group (10.0±5.2 vs. 54.0±25.7 Pa, respectively; P=0.035 and 30.3% ± 24.1% vs. 81.3% ± 13.4%, respectively; P=0.015).

**Figure 2:**
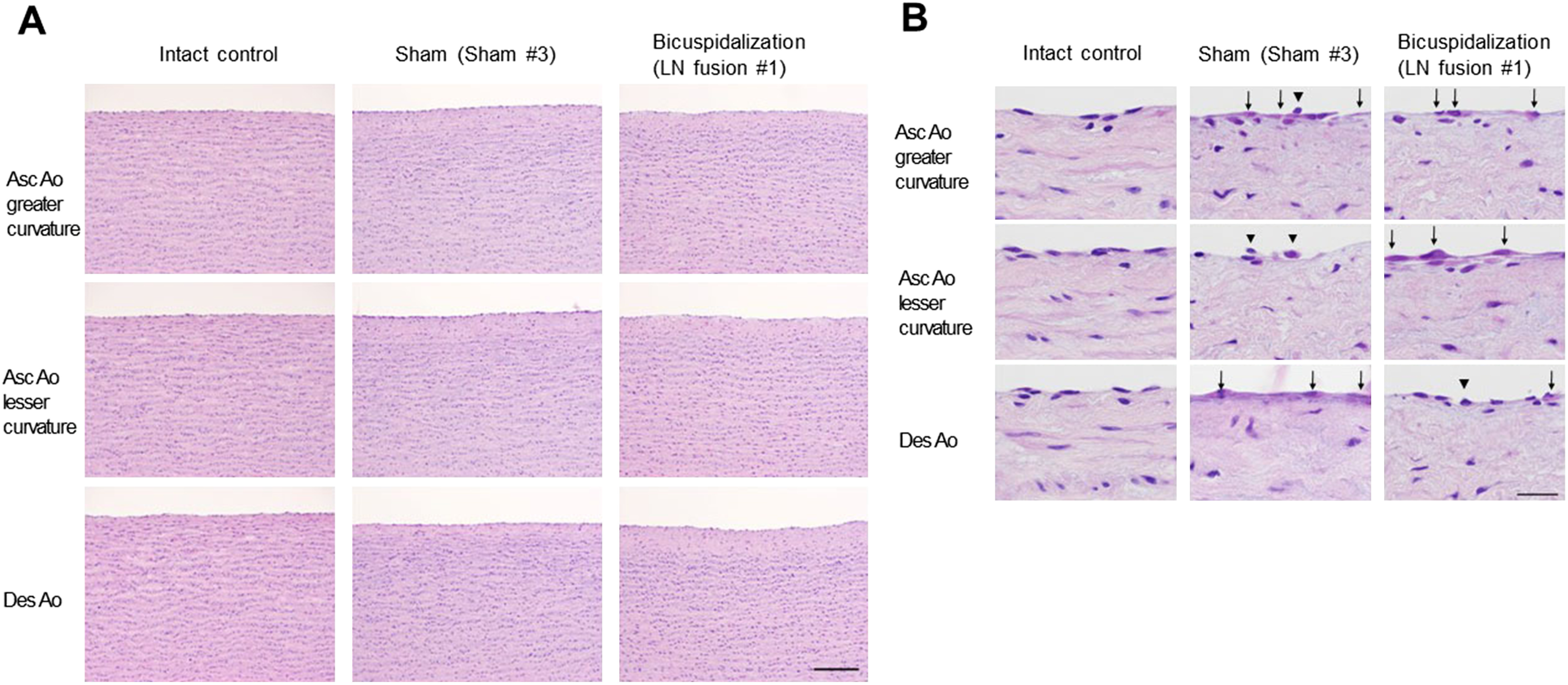
Fig. 2 Representative H&E-stained tissue sections from the greater and lesser curvatures of the proximal ascending and from the proximal descending aorta from the intact control animal (left panel) and animals subjected to sham surgery (middle panel) or bicuspidalization (right panel). The intimal layer is seen in the upper portion of each image. (**A**) Low-power field imaging shows subintimal edema in all three aortic areas of animals subjected to sham surgery and those subjected to bicuspidalization surgery. Bar=100 µm. (**B**) High-power-field imaging shows an irregular endothelial covering, with degeneration of endothelial cells in all three aortic areas of animals subjected to either sham surgery or bicuspidalization. Arrows: degenerated endothelial cells; Arrowheads: detached endothelial cells. Bar=10 µm.

Light microscopy examination of H&E-stained sections revealed the following: in the intact control animal, the aortic walls were of a normal three-layer structure, with the innermost layer covered in endothelial cells. In contrast, in the sham surgery and bicuspidalization group animals, low-power field imaging showed focal subintimal edema in all three areas from which tissue specimens were obtained, including the greater and lesser curvature of the ascending aorta, and descedning aorta (**Fig. 2A**). High-power field imaging of tissues obtained from all three areas in both the sham surgery and bicuspidalization group animals showed that endothelial cells lined the walls irregularly; there was some endothelial degeneration, including denudation and detachment of endothelial cells (**Fig. 2B**). There were no obvious histologic differences in the aortic wall between the sham surgery and bicuspidalization groups.

H&E: hematoxylin and eosin; LN: left coronary cusp-non coronary cusp; AscAo: ascending aorta; DscAo: descending aorta

**Figure 3:**
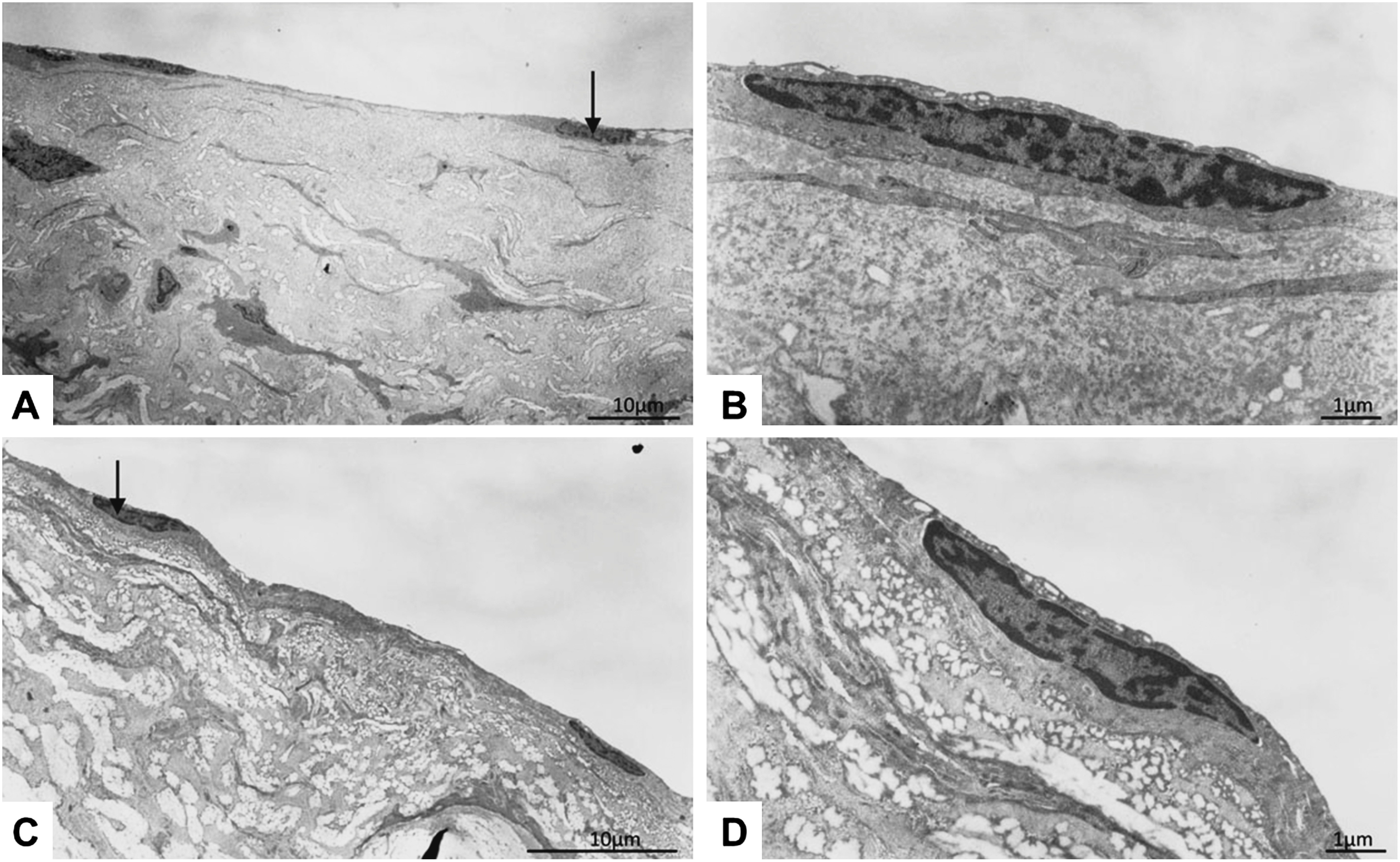
Fig. 3 Transmission electron micrographs of tissues from the ascending (**A**, **B**) and descending aortas (**C**, **D**) of the intact control animal. The endothelial cell marked by an arrow on each of the low-power field images (**A**, **C**) is also marked by an arrow on the corresponding high-power field images (**B**, **D**). Flat endothelial cells with few nuclear deformities are seen in both areas of the thoracic aorta.

**Figure 4:**
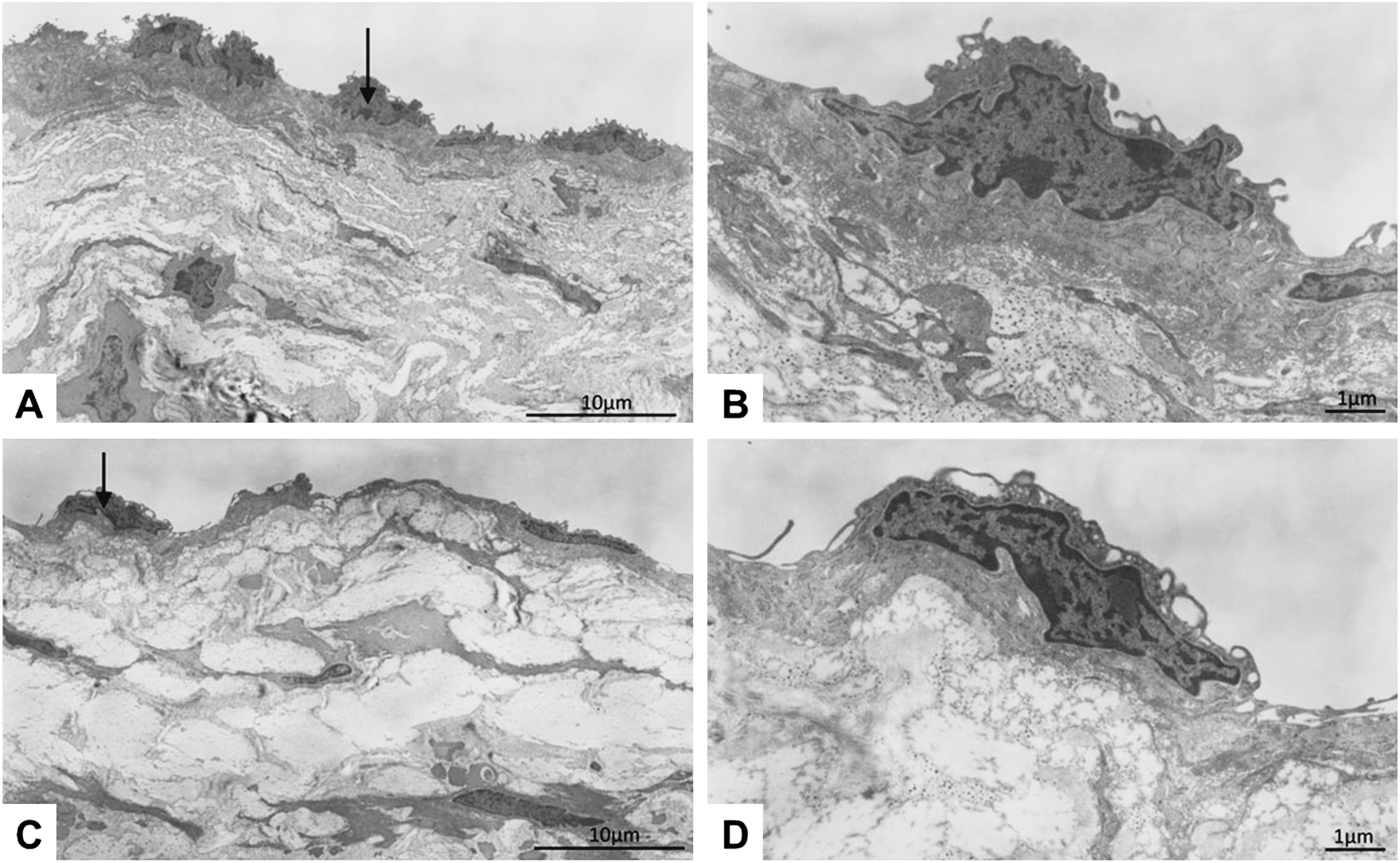
Fig. 4 Transmission electron micrographs of tissues from the ascending (**A**, **B**) and descending aortas (**C**, **D**) of an animal subjected to sham surgery. The endothelial cell marked by an arrow on each of the low-power field images (**A**, **C**) is also marked by an arrow on the corresponding high-power field images (**B**, **D**). Cell swelling, nuclear deformity, and irregularly protruding cell processes on the luminal surface are seen in both areas of the thoracic aorta.

**Figure 5:**
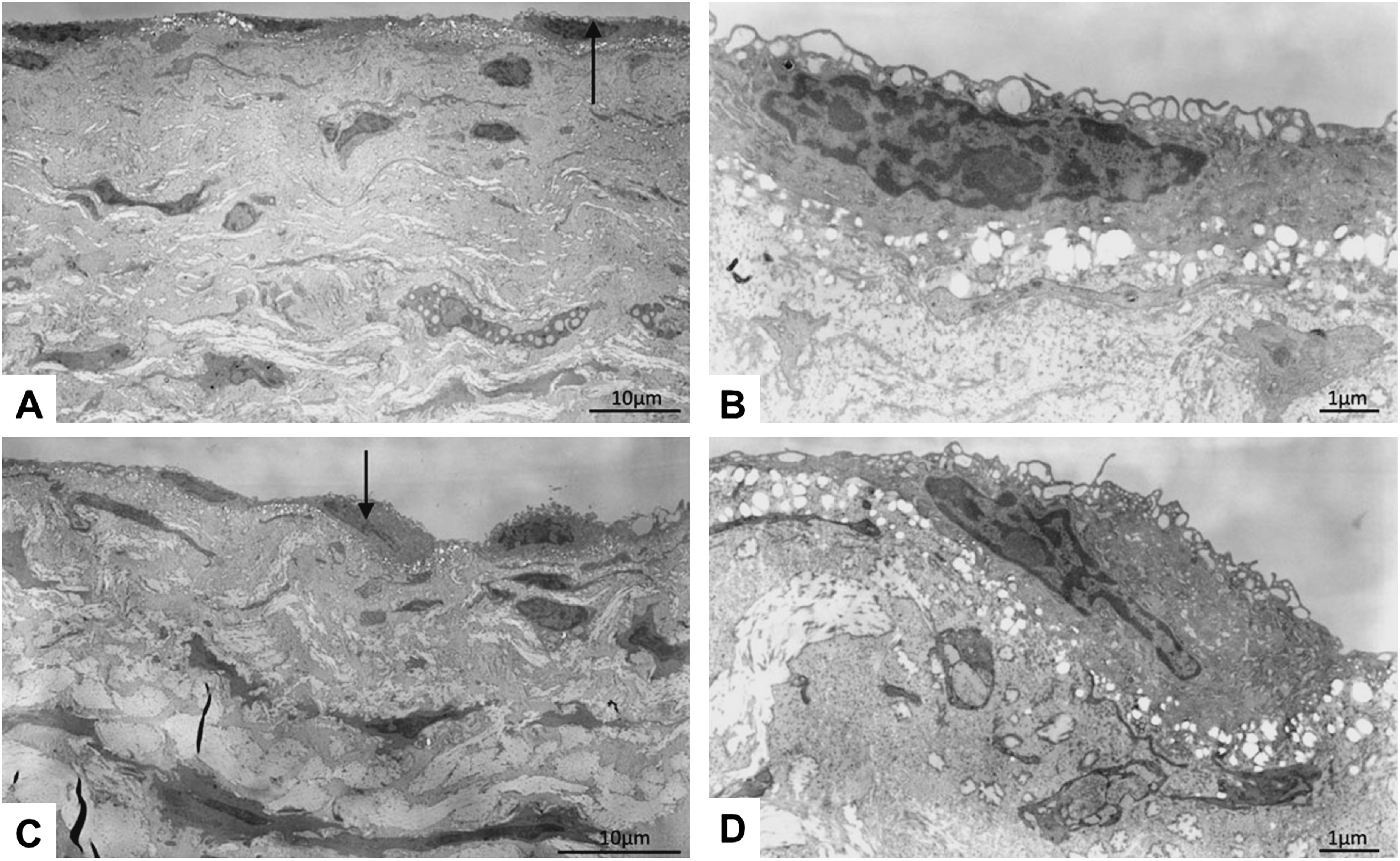
Fig. 5 Transmission electron micrographs of tissues from the ascending (**A**, **B**) and descending aortas (**C**, **D**) of an animal subjected to bicuspidalization. The endothelial cell marked by an arrow on each of the low-power field images (**A**, **C**) is also marked by an arrow on the corresponding high-power field images (**B**, **D**). Cell swelling, nuclear deformity, and irregularly protruding cell processes on the luminal surface are seen in both areas of the thoracic aorta.

Finally, we investigated the morphology of aortic endothelial cells using TEM. Representative TEM images of the greater curvature of the proximal ascending and proximal descending aortas are shown in **Fig. 3**. Flat endothelial cells with few nuclear deformities were seen in both areas of the thoracic aorta of the intact control animal. In contrast, in both the sham and bicuspidalization groups, endothelial cells in the greater curvature of the proximal ascending and proximal descending aortas were characterized morphologically by swelling, nuclear deformity, and irregularly protruding processes on the luminal surface (**Figs. 4** and **5**). TEM examination of the lesser curvature of the proximal ascending aorta yielded findings similar to those of the greater curvature of the proximal ascending and proximal descending aortas in both the sham surgery and bicuspidalization groups (data not shown). In addition, sporadic desquamation of endothelial cells was seen in the thoracic aorta of the sham surgery and bicuspidalization group animals. Thus, both sham surgery and bicuspidalization resulted in endothelial cell damage throughout the thoracic aorta, regardless of WSS distribution. Additionally, no difference in cell damage between the ascending and descending aortas was found in either the sham surgery or bicuspidalization group animals.

## Discussion

Aortic dilatation associated with BAV increases patients’ risk of an aortic event. A retrospective cohort study showed the age-adjusted risk for aortic dissection to be 8.4 times higher in patients with BAV than in the general population.^13)^ Despite recently improved outcomes,^14)^ aortic dissection remains a serious cardiovascular disorder with poor prognosis. According to a recently reported study of patients with BAV, aortic dissection can occur even in those with a mildly dilated aorta; predissection ascending aorta diameters were <5.0 cm in 76% of patients who suffered type A aortic dissection.^15)^ Therefore, elucidating the mechanisms at play in the early stage of aortic dilatation is important if new biomarkers or drugs for bicuspid aortopathy are to be developed. In this study, we established a porcine in vivo model of bicuspid aortopathy so that we could investigate early histologic change in the aortic wall exposed to eccentric blood flow due to a BAV. To the best of our knowledge, this is the first large animal in vivo model of bicuspid aortopathy. The surgeries (including the sham surgery) were performed safely, with the single postoperative death (13% mortality) attributed to the induction of anesthesia for postsurgical imaging studies. CFD analysis revealed that bicuspidalization increased WSS in the ascending aorta; however, histologic examination showed no obvious endothelial injury or subintimal change in the proximal ascending aorta where the WSS was increased.

Until our study, the bicuspidalization technique had not been used to create an in vivo model for the study of BAV. In vivo experiments have been performed in rodents, including two spontaneous BAV hamster strains and 27 genetically modified mouse strains.^16)^ Representative rodent strains include *Notch1*^−/−^ mice^17)^ and *Nos3*^−/−^ mice (27% incidence of BAV).^18)^ Dilatation of the ascending aorta was shown to develop during early adulthood in *Notch1*^+/−^^8)^ and *Nos3*^−/−^ mice.^9)^ In addition to in vivo rodent models, a few ex vivo large animal models have been reported. Zhu et al. identified a rare congenital porcine BAV with type 0 lateral morphology and studied the valvular hemodynamics using an ex vivo left heart simulator.^11)^ Juraszek et al. performed bicuspidalization in domestic pigs to assess the influence of hemodynamic stress caused by eccentric flow jets.^19)^ Although their model provided for assessment of hemodynamics at an early disease stage, the model was limited for ex vivo experimentation because of flow jets created by non-pulsatile flow and unsuitableness for histopathologic investigations.^19)^

Both intrinsic vulnerability of the aortic wall (genetic factor) and increased hemodynamic stress caused by eccentric flow jets created by the abnormal valve anatomy (hemodynamic factor) are considered to contribute to development of aortopathy.^3,4,20)^ Several genes have been associated with bicuspid aortopathy. McKellar et al. showed *NOTCH1* gene variants in 10% of patients with bicuspid aortopathy vs. 2.1% in control subjects.^5)^ Other genes found to be related to aneurysm formation in patients with BAV include *FBN1*, *ACTA2*, and *TGFBR1* and *TGFBR2*.^21–23)^ Aside from genetic predisposition, asymmetric histopathologic changes in a dilated ascending aorta in patients with BAV supports the hypothesis that hemodynamic stress contributes to development of bicuspid aortopathy.^6)^ Guzzardi et al. reported medial elastin degradation and increased matrix metalloproteinase and transforming growth factor-β expression in regions of high WSS in the dilated ascending aorta.^7)^

Our CFD simulation confirmed that bicuspidalization increased WSS in the ascending aorta. However, no obvious histologic differences were observed between the sham surgery (low WSS) and bicuspidalization (high WSS) groups. Several factors might have contributed to this phenomenon. First, we set the bicuspid flow exposure time to 48 h. This short exposure time was set so that we could focus on very early histologic changes to aortic tissue and also because we realized that postoperative complications, such as respiratory failure, pneumonia, and heart failure, might affect systemic or local inflammatory responses in animals subjected to surgery. However, such a short exposure time might have resulted in minimal endothelial damage in the high WSS area. Second, echocardiography showed a 52% reduction in the aortic valve area after bicuspidalization, which was similar to hemodynamically significant reduction in valve area that defines mild BAV stenosis. Creating a narrower valve area and thus more severe aortic stenosis might provide sufficient hemodynamic stress to cause endothelial cell damage in the aortic wall. Third, the effects of extracorporeal circulation on endothelial cells lining the entire thoracic aorta might have masked the effects of local hemodynamic stress created by bicuspidalization. We observed focal endothelial desquamation and degenerated endothelial cells throughout the thoracic aorta in both the sham surgery and bicuspidalization groups. Extracorporeal circulation has been reported to negatively affect homeostasis of endothelial cells. Schmid et al. reported increased numbers of circulating endothelial and apoptotic endothelial cells in patients who underwent on-pump arrested-heart coronary artery bypass grafting rather than off-pump coronary artery bypass grafting.^24)^ Using an ex vivo experimental model of CPB, Nguyen et al. showed that short-term exposure (≤5 h) to continuous non-pulsatile flow (vs. pulsatile flow) activated pro-inflammatory/pro-angiogenic signaling in cultured human aortic endothelial cells.^25)^ Understanding endothelial cell recovery after damage induced by extracorporeal circulation is important to understand the effects of hemodynamic stress after bicuspidalization.

Our study was limited, first, by its execution as an animal study of small size. The small size is typical of animal studies, but the limited number of samples prevented comparative quantitative histologic examination between animals subjected to sham surgery and those subjected to bicuspidalization. In addition, we did not compare WSS between LN and RN bicuspidalization. Second, we selected 48-hour blood flow exposure, but a longer exposure time is necessary to assess subacute changes in the aortic wall exposed to blood flow after bicuspidalization. Thus, large-scale chronic experiments should be performed in the future. Miniature pigs are suitable for chronic experiments because weight gain during a long experimental period is not problematic, with a mature miniature pig weighing only 40–50 kg.^26)^ Third, our study lacked investigation into the effect of genetic factors on the development of bicuspid aortopathy. Fourth, we were unable to incorporate echocardiography data into our CFD analysis for evaluation of WSS. We hope to develop a means by which echocardiography data can be incorporated into CFD simulation.

## Conclusion

We developed a new in vivo BAV model for examination of bicuspid aortopathy. Bicuspidalization was performed in pigs under CPB support. CFD analysis showed increased WSS in the ascending aorta. The endothelial cell damage observed in pigs subjected to sham surgery and in those subjected to bicuspidalization might have been associated with the non-pulsatile flow during CPB. Further investigation that includes a long follow-up period is needed to examine the influence of BAV-derived hemodynamic stress on the development of aortopathy.

## References

[1] Sillesen AS, Vøgg O, Pihl C, et al. Prevalence of bicuspid aortic valve and associated aortopathy in newborns in Copenhagen, Denmark. JAMA 2021; 325: 561-7. 33560321 10.1001/jama.2020.27205 PMC7873775

[2] Li Y, Wei X, Zhao Z, et al. Prevalence and complications of bicuspid aortic valve in Chinese according to echocardiographic database. Am J Cardiol 2017; 120: 287-91. 28532768 10.1016/j.amjcard.2017.04.025

[3] Verma S, Siu SC. Aortic dilatation in patients with bicuspid aortic valve. N Engl J Med 2014; 370: 1920-9. 24827036 10.1056/NEJMra1207059

[4] Siu SC, Silversides CK. Bicuspid aortic valve disease. J Am Coll Cardiol 2010; 55: 2789-800. 20579534 10.1016/j.jacc.2009.12.068

[5] McKellar SH, Tester DJ, Yagubyan M, et al. Novel NOTCH1 mutations in patients with bicuspid aortic valve disease and thoracic aortic aneurysms. J Thorac Cardiovasc Surg 2007; 134: 290-6. 17662764 10.1016/j.jtcvs.2007.02.041

[6] Della Corte A, Quarto C, Bancone C, et al. Spatiotemporal patterns of smooth muscle cell changes in ascending aortic dilatation with bicuspid and tricuspid aortic valve stenosis: focus on cell-matrix signaling. J Thorac Cardiovasc Surg 2008; 135: 8-18. 18.e1-2. 18179910 10.1016/j.jtcvs.2007.09.009

[7] Guzzardi DG, Barker AJ, van Ooij P, et al. Valve-related hemodynamics mediate human bicuspid aortopathy: insights from wall. J Am Coll Cardiol 2015; 66: 892-900. 26293758 10.1016/j.jacc.2015.06.1310 PMC4545965

[8] Koenig SN, LaHaye S, Feller JD, et al. Notch1 haploinsufficiency causes ascending aortic aneurysms in mice. JCI Insight 2017; 2: e91353. 10.1172/jci.insight.91353 PMC5752295 29093270

[9] Peterson JC, Wisse LJ, Wirokromo V, et al. Disturbed nitric oxide signalling gives rise to congenital bicuspid aortic valve and aortopathy. Dis Model Mech 2020; 13: dmm044990. 32801116 10.1242/dmm.044990 PMC7541347

[10] Visser LC, Scansen BA. Congenital bicuspid aortic valve in an English bulldog. J Vet Cardiol 2013; 15: 87-92. 23434244 10.1016/j.jvc.2012.12.001

[11] Zhu Y, Imbrie-Moore AM, Park MH, et al. Ex vivo analysis of a porcine bicuspid aortic valve and aneurysm disease model. Ann Thorac Surg 2021; 111: e113-5. 32663472 10.1016/j.athoracsur.2020.05.086 PMC8357412

[12] Kimura N, Nakamura M, Komiya K, et al. Patient-specific assessment of hemodynamics by computational fluid dynamics in patients with bicuspid aortopathy. J Thorac Cardiovasc Surg 2017; 153: S52-S62.e3. 28190607 10.1016/j.jtcvs.2016.12.033

[13] Michelena HI, Khanna AD, Mahoney D, et al. Incidence of aortic complications in patients with bicuspid aortic valves. JAMA 2011; 306: 1104-12. 21917581 10.1001/jama.2011.1286

[14] Shimizu H, Okada M, Toh Y, et al. Thoracic and cardiovascular surgeries in Japan during 2018: annual report by the Japanese Association for Thoracic Surgery. Gen Thorac Cardiovasc Surg 2021; 69: 179-212. 33090365 10.1007/s11748-020-01460-w PMC7788037

[15] Kreibich M, Rylski B, Czerny M, et al. Type A aortic dissection in patients with bicuspid aortic valve aortopathy. Ann Thorac Surg 2020; 109: 94-100. 31265822 10.1016/j.athoracsur.2019.05.022

[16] Fernández B, Soto-Navarrete MT, López-García A, et al. Bicuspid aortic valve in 2 model species and review of the literature. Vet Pathol 2020; 57: 321-31. 32079504 10.1177/0300985819900018

[17] Garg V, Muth AN, Ransom JF, et al. Mutations in NOTCH1 cause aortic valve disease. Nature 2005; 437: 270-4. 16025100 10.1038/nature03940

[18] Peterson JC, Chughtai M, Wisse LJ, et al. Bicuspid aortic valve formation: Nos3 mutation leads to abnormal lineage patterning of neural crest cells and the second heart field. Dis Model Mech 2018; 11: dmm034637. 30242109 10.1242/dmm.034637 PMC6215433

[19] Juraszek A, Dziodzio T, Stoiber M, et al. The influence of bicuspid aortic valves on the dynamic pressure distribution in the ascending aorta: a porcine ex vivo model. Eur J Cardiothorac Surg 2014; 46: 349-55; discussion, 355. 24644312 10.1093/ejcts/ezu055

[20] Messner B, Bernhard D. Bicuspid aortic valve-associated aortopathy: Where do we stand? J Mol Cell Cardiol 2019; 133: 76-85. 31152748 10.1016/j.yjmcc.2019.05.023

[21] Attias D, Stheneur C, Roy C, et al. Comparison of clinical presentations and outcomes between patients with TGFBR2 and FBN1 mutations in Marfan syndrome and related disorders. Circulation 2009; 120: 2541-9. 19996017 10.1161/CIRCULATIONAHA.109.887042

[22] Guo DC, Pannu H, Tran-Fadulu V, et al. Mutations in smooth muscle alpha-actin (ACTA2) lead to thoracic aortic aneurysms and dissections. Nat Genet 2007; 39: 1488-93. 17994018 10.1038/ng.2007.6

[23] Arrington CB, Sower CT, Chuckwuk N, et al. Absence of TGFBR1 and TGFBR2 mutations in patients with bicuspid aortic valve and aortic dilation. Am J Cardiol 2008; 102: 629-31. 18721526 10.1016/j.amjcard.2008.04.044

[24] Schmid FX, Vudattu N, Floerchinger B, et al. Endothelial apoptosis and circulating endothelial cells after bypass grafting with and without cardiopulmonary bypass. Eur J Cardiothorac Surg 2006; 29: 496-500. 16504531 10.1016/j.ejcts.2006.01.029

[25] Nguyen KT, Donoghue L, Giridharan GA, et al. Acute response of human aortic endothelial cells to loss of pulsatility as seen during cardiopulmonary bypass. Cells Tissues Organs 2021; 25: 1-11. 10.1159/000512558 33631743

[26] Kobayashi E, Hishikawa S, Teratani T, et al. The pig as a model for translational research: overview of porcine animal models at Jichi Medical University. Transplant Res 2012; 1: 8. 23369409 10.1186/2047-1440-1-8 PMC3560993

