# Supplementary material for: Bicuspidalization of the Native Tricuspid Aortic Valve: A Porcine in Vivo Model of Bicuspid Aortopathy
Source: Ann Vasc Dis. 2022 Mar 25;15(1):37–44. doi: 10.3400/avd.oa.21-00116 (PMC8958402; doi:10.3400/avd.oa.21-00116)
Supplement: Supplementary Data [file avd-15-1-oa.21-00116-s001.pdf]

Supplementary Materials

Materials and Methods

CFD simulation of blood flow

Anatomic geometry was determined from CT angiograms. CT angiography was performed with a CT scanner (SOMATOM Definition AS+ [128-slice], Siemens Healthcare GmbH, Erlangen, Germany) under general anesthesia. For contrast enhancement, a 600-mg iodine/kg bolus of iopamidol was administered intravenously through the ear vein. The resulting CT angiograms had a spatial resolution of 0.7812 mm x 0.7812 mm x 1 mm. The intraluminal region of interest was reconstructed with use of commercially available imaging software AMIRA (ver. 5.4.2, Maxnet, Tokyo, Japan), and the surface was smoothed with a commercially available digital sculpting program, 3DCoat (ver. 4.1.17D, Pilgway, Kiev, Ukraine). A virtual bicuspid valve orifice was created with computer-aided design software, Creo Parametric (PTC, Needham, Mass) and applied at the level of the sinotubular junction. For each animal, the bicuspid valve orientation and shape of the orifice were determined echocardiographically and intraoperatively.

Flow rates at various time points in the cardiac cycle were measured at the sinotubular junction and supra-aortic branches in each animal by means of 2D cine phase contrast-MRI. The MRI was performed on a 1.5-T MRI (MAGNETOM ESSENZA; Siemens Healthcare, Erlangen, Germany). Standard clinical MRI settings were used: repetition time, 33 (43) ms; echo time, 5.4 to 6.0 (2.5) ms; velocity encoding range, 300 (500) cm/s; flip angle, 30 (30); slice thickness, 5 (10) mm; matrix, 192 3 192 (256 3 256); and field of view 32 3 24 (30 3 30) cm. All measurements were made under general anesthesia. The R wave on electrocardiogram was used to trigger the MRI acquisition. The flow rate at each time point was calculated by integration of axial velocities within a manually chosen vessel lumen of interest. A moving average was used to smooth the flow rate data, and a cubic spine was used for interpolation.

The surface was covered by five layers of prism meshes, and the rest of the region was filled with tetrahedral meshes. Blood was assumed to be an incompressible Newtonian fluid with a density of

1.06 x 10^3 kg/m^3 and a dynamic viscosity of 3.0 x 10^{-3} Pa/s. Hemodynamics was simulated with a commercially available CFD program (SCRYU ver. 14, Cradle Co, Tokyo, Japan), which incorporates a finite volume method for formulation and the SIMPLEC (semi-implicit method for pressure linked equations-consistent) scheme to couple the equations governing the flow, the Navier-Stokes and continuity equations. No turbulence model was used. The first-order implicit scheme was used for time marching, and the second-order MUSCL (Monotonic Upstream-Centered Scheme for Conservation Laws) cwas used to discretize convective terms. As boundary conditions, the measured and interpolated flow rates were given at the aortic branches and the ascending aorta (the sinotubular junction). A nonslip condition was applied at the wall, with the wall assumed to be rigid. A zero pressure condition was imposed at the end of the descending aorta. The flow simulation was carried out over four cardiac cycles to obtain cyclically repeatable flow patterns.

Supplemental Table 1. Basic characteristics, operative variables, and surgical outcomes of the 8 pigs subjected to surgery and per group

	Overall (n=8)	Sham Surgery (n=3)	Bicuspidalization (n=5)	P value
Body weight (kg)	44.1±4.3	42.9±3.4	44.8±4.9	0.57
Preoperative LVEF (%)	76.5±8.8	75.6±5.9	77.2±11.4	0.84
Postoperative LVEF (%)	61.9±10.9	75.6±5.9	57.2±5.2	0.21
Operation time (min)	251±34	238±40	258±32	0.47
CPB time (min)	82±19	79±20	84±21	0.72
Myocardial ischemia time (min)	62±43	41±18	44±12	0.80
Amount of blood collected by the cell-saver device (mL)	1557±1012	1218±566	1760±1223	0.51
Successful weaning from CPB	8 (100%)	3 (100%)	5 (100%)	1.0
Intraoperative death	0 (0%)	0 (0%)	0 (0%)	1.0
Neurological complication	0 (0%)	0 (0%)	0 (0%)	1.0
Death within 48 hours of surgery*	1 (13%)	0 (0%)	1 (20%)	1.0
Preoperative AVA (cm^2)**	NA	Not measured	2.52±1.15	NA
Postoperative AVA (cm^2)**	NA	Not measured	1.21±0.48	NA

Mean ± standard deviation values or number (and percentage) of animals are shown.

*One animal died of respiratory failure during anesthesia induction for imaging studies 48 hours after the surgery. **Pre- and postoperative AVA were not assessed for the animal in the bicuspidalization group that died. LVEF: left ventricular ejection fraction; CPB: cardiopulmonary bypass; AVA: aortic valve area; NA: not applicable.

Supplemental Fig. 1.

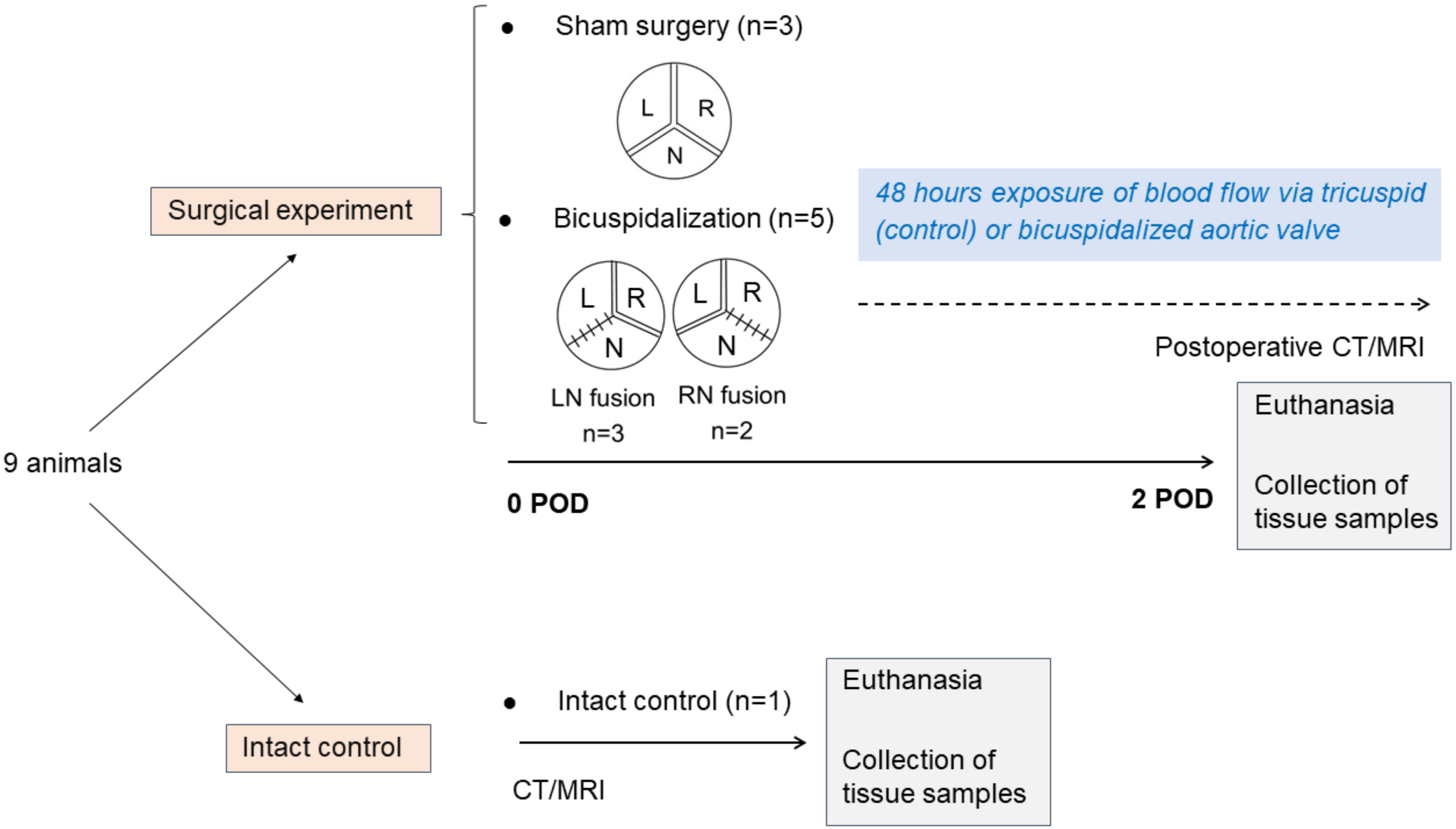

Study protocol prior to CFD analysis. CT and MRI study were performed 48 hours after the surgical procedure. After 48-hour exposure of the tricuspid aortic valve (sham surgery group), or bicuspid aortic valve (bicuspidalization group), animals in the two groups were euthanized, and aortic tissue specimens were taken for histologic examination. Tissues were also obtained from the intact control animal after euthanasia. CT: computed tomography; MRI: magnetic resonance image; POD: postoperative day.

Supplemental Video 1.

Echocardiography of LN type bicuspidalization. A fused left coronary cusp and non coronary cusp by bicuspidalization procedure resulted in a decreased aortic valve opening area.
